# *Bifidobacterium animalis*: the missing link for the cancer-preventive effect of *Gynostemma pentaphyllum*

**DOI:** 10.1080/19490976.2020.1847629

**Published:** 2020-11-24

**Authors:** Weilin Liao, Imran Khan, Guoxin Huang, Shengshuang Chen, Liang Liu, Wai Kit Leong, Xiao Ang Li, Jianlin Wu, W. L. Wendy Hsiao

**Affiliations:** State Key Laboratory of Quality Research in Chinese Medicine, Macau University of Science and Technology, Taipa, Macau SAR

**Keywords:** *Gynostemma pentaphyllum* saponin, *Bifidobacterium animalis*, probiotics, prebiotic, cancer prevention, colonic cancer, Apc*^Min/+^* mice

## Abstract

Colorectal cancer (CRC) ranks the third most common cancer type in both men and women. Besides the known genetic and epigenetic changes in the gut epithelial cells, we now know that disturbed gut microbes could also contribute to the onset and progression of CRC. Hence, keeping a balanced gut microbiota (GM) has become a novel pursue in the medical field, particularly in the area of gastrointestinal disorders. *Gynostemma pentaphyllum* (Gp) is a dietary herbal medicine. In our previous study, Gp saponins (GpS) displayed prebiotic and cancer-preventive properties through the modulation of GM in Apc*^Min/+^* mice. However, the specific group(s) of GM links to the health effects of GpS remains unknown. To track down the missing link, we first investigated and found that inoculation with fecal materials from GpS-treated Apc*^Min/+^* mice effectively reduces polyps in Apc*^Min/+^* mice. From the same source of the fecal sample, we successfully isolated 16 bacterial species. Out of the 16 bacteria, *Bifidobacterium animalis* stands out as the responder to the GpS-growth stimulus. Biochemical and RNAseq analysis demonstrated that GpS enhanced expressions of a wide range of genes encoding biogenesis and metabolic pathways in *B. animalis* culture. Moreover, we found that colonization of *B. animalis* markedly reduces the polyp burden in Apc*^Min/+^* mice. These findings reveal a mutualistic interaction between the prebiotic and a probiotic to achieve anticancer and cancer-preventive activities. Our result, for the first time, unveils the anticancer function of *B. animalis* and extend the probiotic horizon of *B. animalis*.

## Introduction

Colorectal cancer is the third most common cancer type in the world.^1,2^ Most of the cases are the results of sporadic accumulations of gene mutations and epigenetic modifications. The initial mutation often occurs in the tumor suppressor gene, adenoma polyposis coli (*APC*), which encodes a multifunctional protein in the WNT signaling pathway. Mutated *APC* gene drives the formation of benign adenomas. The subsequent mutations in *KRAS* oncogene and p53 tumor suppressor gene transform adenomas to malignancy. Recent studies showed that *APC* restoration could revert cancerous tumors to functioning normal cells, further emphasizing the critical role of *APC* in colonic cancer.^3,4^

Besides the impact of gene mutations, there are other risk factors involved in the process of colonic carcinogenesis. Among which are the behaviors of the trillion microbes residing in the gut of the host.^5^ It is well recognized that gut microbiota (GM) is an integral part of host physiology and plays a pivotal role in the metabolism and immune system. GM contributes to the protection against the opportunistic pathogens and maintenance of food tolerance and metabolic balance of the host.^6,7^ On the other hand, disruption of the homeostasis of the microbial community could lead to neurological diseases, metabolic, cardiovascular diseases, and gastrointestinal disorders.^8–11^ Among these ailments, significant research has focused on the role of GM in CRC progression.^12–14^ In general, CRC patients harbor lower GM diversity and less abundance of beneficial bacteria.^15^ Several pathogenic microbes in the gut, such as *Fusobacterium* spp., *Streptococcus bovis, Bacteroides fragilis, Peptostreptococcus* spp., and *Porphyromonas* spp. have been associated with the development of CRC.^16–20^ Restoration of the disturbed GM to homeostasis has become a new pursue in CRC treatments. One of the emerging approaches is introducing specific microbes or fecal materials from a healthy donor to the intestinal tract of a recipient patient to change the dysfunctional GM.^21^ Indeed, fecal microbiota transplantation (FMT) has successfully treated recurrent *Clostridium difficile* infection in the clinic.^22^ The other strategy is through the dietary intervention of GM compositions, in particular, the use of bioactive natural products possessing prebiotic property.

Early researches on prebiotics were mainly focusing on plant foods that contain inulin, polyphenols, fructo- or galactooligosaccharides. More recently, certain functional foods, such as *Geranium dielsianum* tea, cassava bagasse flour, and kiwifruit pectins, have also been reported for their prebiotic effects.^23–25^ *Gynostemma pentaphyllum* is a dietary herbal medicine known for its many health benefits. In our previous studies, we demonstrated that triterpenoid saponins from *Gynostemma pentaphylum* together with ginseng, rotoginseng display prebiotic-like effects in the normal mouse model.^26^ Further research showed that *G. pentaphylum* saponin (GpS) exerts significant cancer-preventive effects in Apc*^Min/+^* mice through modulating the GM composition and the gut epithelial microenvironment.^27–29^ We hypothesize that treatment with GpS might have preserved a particular group(s) of bacteria that provides health advantage to the host. In this current study, we performed both *in vivo* and *in vitro* experiments to track down the potential bacterial group(s) that confers the prebiotic and cancer-preventive effect of GpS. We provide evidence that *Bifidobacterium animalis* might be the critical gut microbe contributing to the anticancer and cancer-preventive activities of GpS against colonic cancer.

## Materials and methods

### Herbal source

GpS was purchased from the Hui Zhou Shi Orient Plant Health Care SCL & Tech. CO, Ltd., China. GpS was authenticated and chemically profiled according to the procedure outlined by Wu et al.^30^

### Animals maintenance

C57BL/6J and Apc*^Min/+^* mice were originally purchased from Jackson Laboratory and bred inhouse for the experiments. Mice were maintained in IVC cages and kept in a 12-h/12-h dark-light cycle at 20–22 °C and 40–60% humidity with free access to food and water. All the mice were fed with PicoLab®Rodent Diet 20-5053 (LabDiet, USA).

### Fecal sample preparation and fecal microbial transplant (FMT)

**Figure 1.**
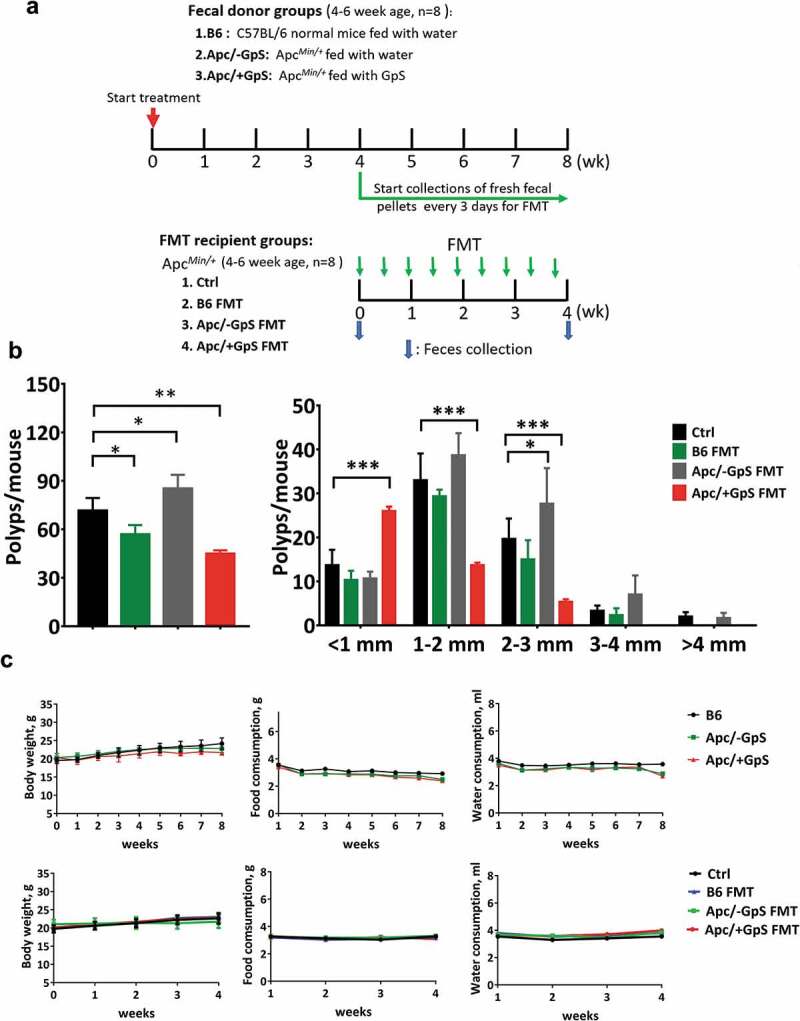
Fecal transplant from GpS-treated Apc*^Min/+^* mice reduced the number and the size of polyps in Apc*^Min/+^* mice. (a) FMT treatment scheme. (b) The total number and the size distribution of the polyps. (c) Body weight, food and water consumptions of the FMT donor mice and the FMT recipient mice. The number was significantly (p ≤ 0.01) lower in the mice treated with Apc/+GpS FMT group (polyp = 45 ± 2), B6 FMT group (polyp = 57 ± 5.57) also significantly (p ≤ 0.05) lower with control group. Interestingly, the Apc/-GpS FMT group (polyp = 85.33 ± 8.39) higher than control. Data is presented as mean ± SD, n = 8. Statistically significance was calculated with One-way ANOVA with Dunnett’s Post Hoc multiple comparison. *p ≤ .05, **p ≤ .01, ***p ≤ .001

For the FMT experiment, we first set up 16 Apc*^Min/+^* mice (aged 4–6 weeks, male) that were randomly divided into GpS treatment (designated as Apc/+GpS) and non-treatment groups (designated as Apc/-GpS). In parallel, eight C57BL/6j mice (aged 4–6 weeks, male) were fed with water as the wildtype control, designated as B6 group. Mice from the Apc/+GpS group were gavage daily with 300 mg/kg of GpS dissolved in 200 μl sterile water, while mice from the Apc/-GpS and the B6 groups were fed with 200 μl water. The treatments were carried out for 8 weeks (Figure 1a). At the end of the 4th week, 200 mg/mouse of fresh fecal pellets were collected every 3 d and used for the FMT experiment. The fecal pellets from 8 mice in each group were combined, homogenized in 5 ml ice-cold sterile water, and vortexed for 3 min. The fecal mixture was centrifuged at 1000 g for 5 min at 4°C. The resulting supernatant was used freshly for FMT. For the FMT, a total of 32 Apc*^Min/+^* mice (aged 4–6 weeks, male) from different litters were randomly divided into 4 groups, i.e., the Ctrl (no FMT), B6 FMT (fecal samples from the normal C57BL/6j donor mice), Apc/-GpS FMT (fecal sample from the Apc/-GpS donor mice), and Apc/+GpS FMT (feces from the Apc/+GpS donor mice). Except for the Ctrl group, all mice received fecal transplants (200 μl) every 3rd day for four consecutive weeks, as illustrated in Figure 1b. During the entire treatment scheme, mice were monitored for body weight, food, and water consumption. At the end of the experiment, mice were euthanized. Intestinal tissues were collected, and colonic polyp counts were performed. All the experimental procedures were performed following the approved ethics guidelines of the “Ethics Review Committee for Animal Research” of the Macau University of Science & Technology, Macau.

### Isolation and identification of bacterial colonies from fecal samples of Apc^Min/+^ mice treated with GpS

Approximately 5 g of stool was collected from the Apc/+GpS mice (Figure 1a) and suspended in 45 mL sterile saline water and centrifuged at 200 rpm at 37 °C for 30 minutes under anaerobic condition. The resulting supernatant was anaerobically suspended, diluted, and spread onto the agar growth medium (Table S4) kept in an anaerobic chamber filled with 5% CO_2_, 10% H_2_, and 85% N_2_ gas (Whitley A35 Workstation, Don Whitley Scientific Limited, UK). The recovered colonies were purified and later processed for identification using MALDI-TOF MS (Bruker Daltonics, Billerica, Mass., USA). Briefly, the individual bacterial colony was smeared on MALDI-TOF target plate and covered with 1 µl matrix solution (500 µl acetonitrile, 25 µl trifluoroacetic acid, 5 mg α-cyano-4- hydroxycinnamic acid in 475 µl HPLC grade water). After drying, the spot was read and evaluated with flexControl 3.0 software. Spectrum with score ≥1.9 was accepted for bacteria identification. A bacterium with a score ≤1.9 was processed further for identification using16S rRNA gene sequencing.

### Fecal DNA extraction and ERIC-PCR analysis

Fecal samples were collected from individual mice and stored at −80 °C for later DNA extraction. Fecal DNA was prepared using the QIAamp DNA Stool Mini Kit (QIAGEN) according to the manufacturer’s instructions. The GM profile was analyzed using ERIC-PCR analysis, as previously described.^27^

### Quantitative real-time PCR (qPCR) analysis

The quantitation of speciﬁc bacteria was measured by qPCR using Applied Biosystems ViiA™ 7 PCR system (Carlsbad, CA, USA) with taxon-speciﬁc 16S rRNA gene primers (Invitrogen, Carlsbad, CA, USA). The specific primer sequences for qPCR were listed in Table S5. The qPCR was carried out as described.^26^ β-actin was used as an internal control to normalize the PCR reaction for each specific marker. The 2-ΔΔCt method was applied to calculate the fold change of relative gene expression. ΔΔCt = (Cttreatment_target gene – Cttreatment_reference gene) – (Ctcontrol_target gene – Ctcontrol_reference gene).

### Bacteria growth curves

Sixteen isolated bacterial species from the fecal preparation of Apc/+GpS mice (Figure 1a) were cultured in the growth medium broth (Table S1). The composition of this medium is based on Dubourg et al.^31^ with slight modification.^31^ Bacteria were cultured in a 96 well microtiter plate and incubated at 37 °C under anaerobic conditions. The dosage effect of GpS was assessed, ranging from 0.01 to 0.3 mg/ml. The microtiter plate was scanned using a Microplate Reader (Tecan Infinite 200 Pro) at 600 μm at 0, 2, 4, 6, 8, 10, 12-h time points.

### RNA extraction and RNA sequencing

Total RNA was isolated from *B. animalis* culture using RiboPure™-Bacteria Kit. RNA samples were resuspended in 30 μl water, and the concentration was determined using the Qubit® RNA Assay Kit (Life Technologies). For RNAseq, 1 µg of total RNA was further clean-up for DNA contamination using Baseline-ZERO™ DNase (Epicenter) following the manufacturer’s instructions. Then, DNA-free RNA samples were used for rRNA removal using RiboMinus™ rRNA Removal Kit (Bacteria; Thermo Fisher Scientific) and followed by a final purification step with RNA Clean & Concentrator^TM^-5 columns (Zymo Research).

KAPA mRNA HyperPrep Kits (Roche) was used for library preparation by following the manufacturer’s instructions. Qubit® dsDNA HS Assay Kit (Life Technologies) was used to measure the final concentration of all the libraries. Agilent 2100 Bioanalyzer (Agilent Technologies) was used to determine the average library size. Equimolar ratios of 0.6 nM of libraries were pooled and sequenced paired-end (2 × 250bp) for 500 cycles using the NovaSeq 6000 system (Illumina).

Forward and reverse fastq files were paired-end joined using QIIME.^32^ Primers and adaptors were trimmed, and any ambiguous reads were removed. Reads with length range from 200 to 251 bp were selected for annotation. SPARTA was used for filter, QC, alignment, and count transcript abundance.^33^ FASTA and GFF file of reference bacterium (*Bifidobacterium animalis* subsp. *animalis* ATCC 25527) were downloaded from the NCBI Genome.

### Data analysis

Network analysis was performed with Cytoscape (3.8.0) by implementing GeneMania app (www.genemania.org). Circos plot was generated using Circa (OMGenomics). Statistical analysis was performed using GraphPad Prism 7. Partial least squares discriminant analysis (PLS-DA) was performed to visualize the microbial community changes before and after treatment (SIMCA-P 14.0, Umetrics, Umea, Sweden) with a confidence level of 95% (*p* < .05).

## Results

### Fecal materials from GpS-treated Apc^Min/+^ mice significantly reduce polyp burden in Apc^Min/+^ mice

In the attempt to tighten the link between the GM and the cancer-preventive properties of GpS, we performed FMT in Apc*^Min/+^* mice with fecal samples derived from the Apc*^Min/+^* mice pre-treated with GpS and compared to those without GpS treatment, and the wildtype control (Figure 1a). The results showed that the control Apc*^Min/+^* mice (without GpS and FMT) and the Apc/-GpS FMT mice developed 71.67 ± 7.77 and 85.33 ± 8.39 polyps, respectively (Figure 1b). We noted that the polyp number in the Apc/-GpS FMT mice is significantly higher than the Apc*^Min/+^* control mice. It is plausible that the presence of potential pathogens in the fecal samples of Apc*^Min/+^* mice might create extra tumor burden to the Apc/-GpS FMT mice. In contrast, the Apc/+GpS FMT mice showed a strikingly low polyp number (45 ± 2) (Figure 1b). Interestingly, the Apc*^Min/+^* mice that received FMT from normal C57BL/6j (B6 FMT group) also showed a substantial drop in polyp number (57 ± 5.57) (Figure 1b). No observable advert effect was recorded in this experiment (Figure 1c,d).

### Fecal transplant improves the microbial profiles of the Apc^Min/+^ mice

**Figure 2.**
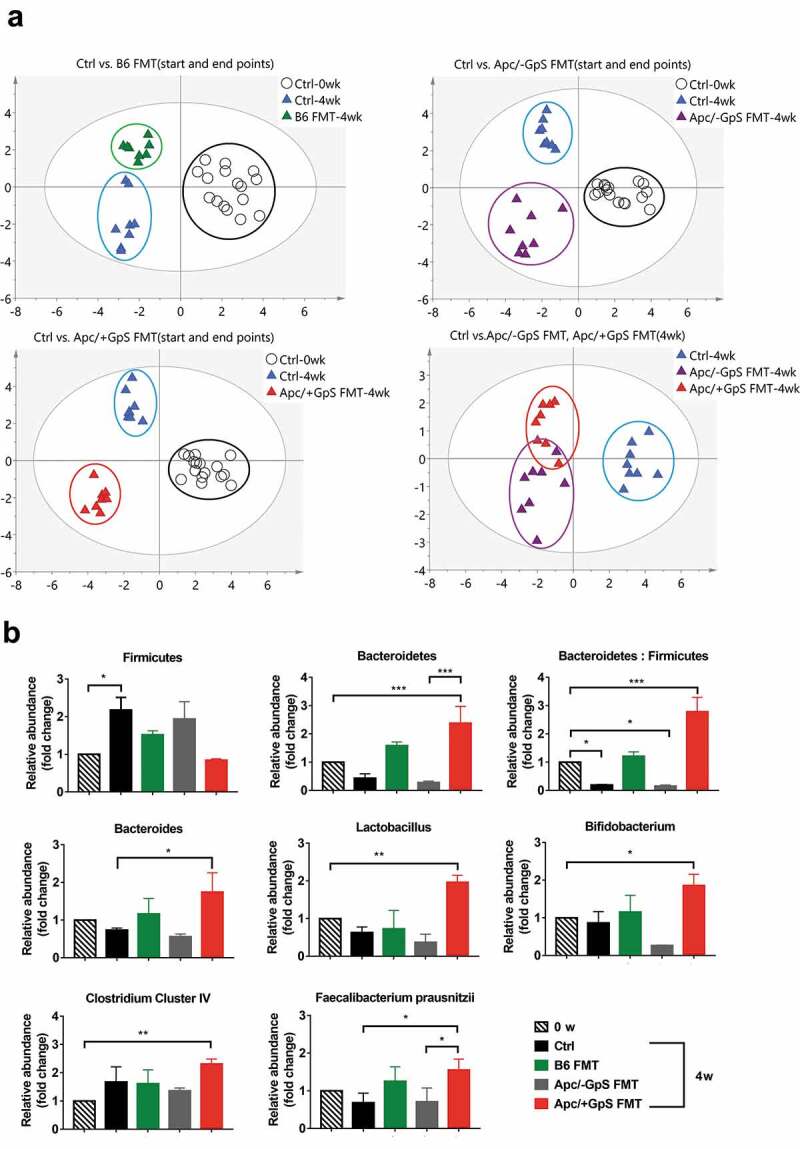
Fecal transplant from GpS-treated Apc*^Min/+^* mice altered GM profiles and enhanced beneficial gut microbes in the Apc*^Min/+^* mice. (a) PLS-DA plots of ERIC-PCR generated data. Each dot represents the bacterial profile of individual mice (n = 8). (b) Distribution of beneficial bacteria in the fecal samples of the FMT mice. Taxon-specific primers were applied and normalized to that of the total bacteria in the qPCR reaction. The data is expressed as fold change over the wk0 control group. Statistically significance was calculated with One-way ANOVA with Dunnett’s Post Hoc multiple comparison. *p ≤ .05, **p ≤ .01, ***p ≤ .001. N = 3

Given the positive results above, we subsequently investigated the GM compositions of the fecal DNA samples of the FMT recipient mice. The fecal samples were collected on week 0 (wk0) and the end of 4th week (wk4) from four experimental groups, i.e., the Ctrl, B6 FMT, Apc/-GpS FMT, and Apc/+GpS FMT groups (Figure 1a). Fecal DNAs were extracted and evaluated for the similarity in GM profile among four groups using ERIC-PCR. Through PLS-DA plots, apparent clustering in the GM profile was noticed among experimental groups (Figure 2a). Besides, longitudinal changes within the same group of mice (wk0 vs. wk4) were also detected (Figure 2a).

In our previous reports,^26,27,29^ treatment with GpS boosted beneficial gut microbes and exerted prebiotic effects in both normal and Apc*^Min/+^* mice. Therefore, in this study, we performed qPCR to survey the abundance of two major gut commensal phyla, i.e., Firmicutes and Bacteroidetes, among the experimental mice. The results showed that after 4 weeks of transplantation, the Apc/+GpS FMT group displayed a significant increment of Bacteroides, as well as the Bacteroides/Firmicutes ratio, compared to the control and the Apc/-GpS FMT groups (Figure 2b). At the same time, we also evaluated common beneficial bacteria at the genera level, including *Bacteroides, Bifidobacterium, Lactobacillus*, and *Clostridium Cluster IV*. We noticed that all the tested bacterial groups were significantly enhanced in the Apc*^Min/+^* mice colonized with fecal samples from the Apc/+GpS mice. Besides, the level of *Clostridium Cluster IV*, which is one of the well-known butyrate-producing bacterial group, was also elevated in the Apc/+GpS FMT group compared to any other treatment group. Moreover, within *Clostridium cluster IV*, the anti-inflammatory symbiotic species *Faecalibacterium prausnitzii* was markedly enhanced in the Apc/+GpS FMT group. It is worth noting that most of the tested beneficial bacteria were also elevated in Apc*^Min/+^* mice colonized with feces derived from the wild type C57BL/6j.

### GpS stimulate the growth of *B. animalis*

**Figure 3.**
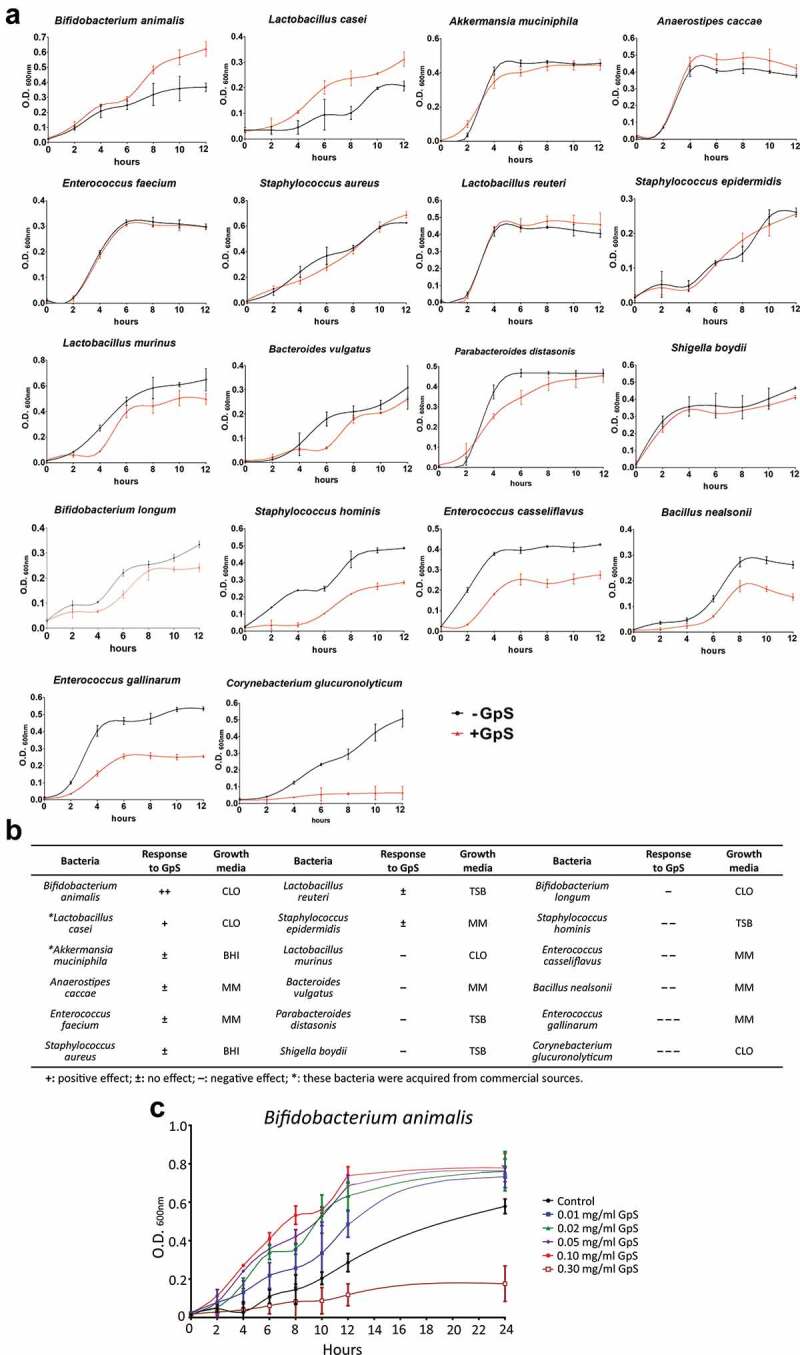
Effects of GpS on the growth of bacteria isolated from the fecal sample of GpS-treated Apc*^Min/+^* mice. (a) Growth curves of bacteria isolates in the presence and absence of 0.10 mg/ml GpS. (b) Summary of the growth responses to GpS shown in panel A. CLO: Clostridium medium; BHI: Brain heart infusion broth; TSB: Tryptic soy broth; MM: Modified growth medium. (c) GpS dosage effects on *B. animalis*. Bacterium was grown in each designated culture medium as indicated in Panel B. The total no of cells was determined at 600 nm using a microplate reader (Tecan Infinite 200 Pro). Data are representative of three independent experiments

To investigate whether the enrichment of beneficial bacteria is due to the direct or indirect growth-stimulating effects of GpS, we cultured the feces solution obtained from the Apc/+GpS mice (Figure 1a) on a modified bacterial agar growth medium under an anaerobic chamber. A total of 16 bacterial species were recovered and identified by MALDI-MS spectrometer. All 16 species were tested for their growth responses to GpS supplement in the growth medium. Interestingly, except *B. animalis*, all the isolated species were either not responsive or even inhibited by the GpS (Figure 3a,b). We further challenged *B. animalis* with GpS ranging from 0.01 to 0.3 mg/ml final concentration. The result showed that except at 0.3 mg/ml, GpS significantly stimulated the growth of *B. animalis* (Figure 3c).

### GpS stimulates the production of short-chain and medium-chain fatty acids in *B. animalis* cultures

**Figure 4.**
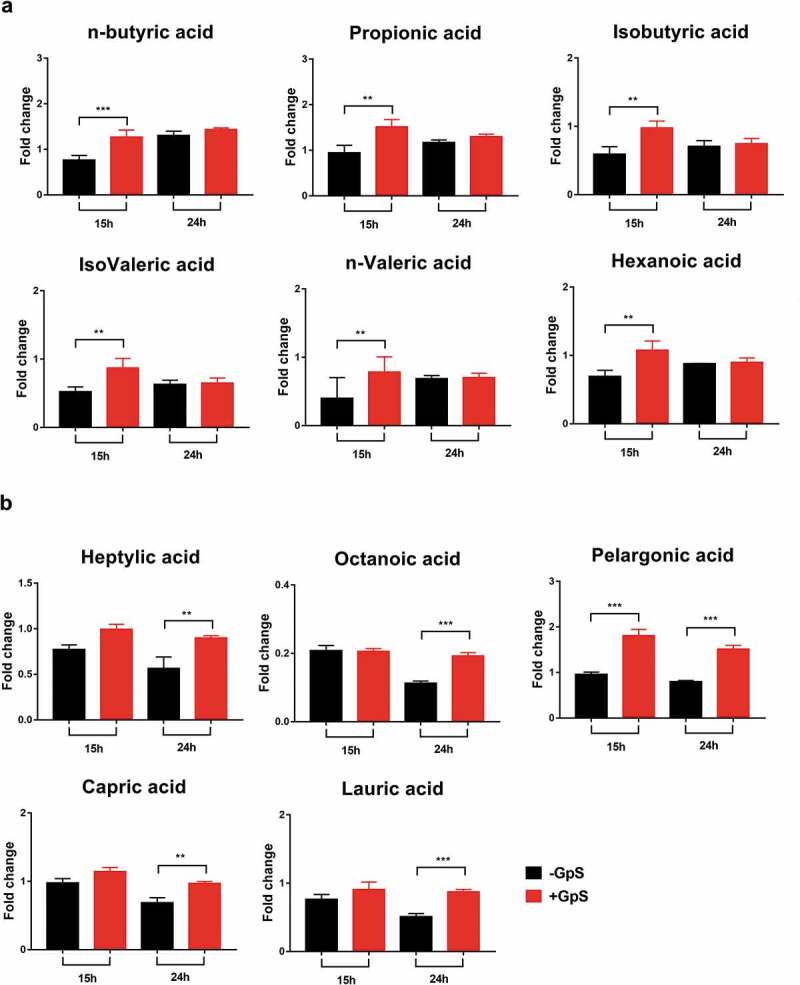
Effects of GpS on the (a) SCFA and (b) MCFA metabolites in *B. animalis* cultures measured at 15th and 24th hour time points. The metabolites were analyzed using UHPLC-Q_TOF/MS. Data is presented as the mean ± SD, n = 3. T test was applied to calculate the statistical significance. *p ≤ .05, **p ≤ .01, ***p ≤ .001

*B. animalis* is a short-chain fatty acid (SCFA) producing bacterium. It is aligned with our previous finding that GpS treatment preferentially enhances SCFA-producing bacteria in mice. Besides growth stimulation, we tested whether GpS could uplift the production of SCFAs and medium-chain fatty acids (MCFAs) of *B. animalis in vitro*. For this purpose, *B. animalis* was grown in growth media supplemented with and without 0.1 mg/ml GpS. The culture media were sampled at 15- and 24-h time points for short-chain and MCFAs analysis using UHPLCQ-TOF/MS. At a 15-h time point, we noticed a higher level of SCFAs metabolites in the presence of GpS compared to the control group (Figure 4a). In contrast, the effect of GpS on MCFAs production was more prominent at the 24-h time point (Figure 4b).

### GpS enhances expressions of genes regulating biogenesis and metabolic pathways

**Figure 5.**
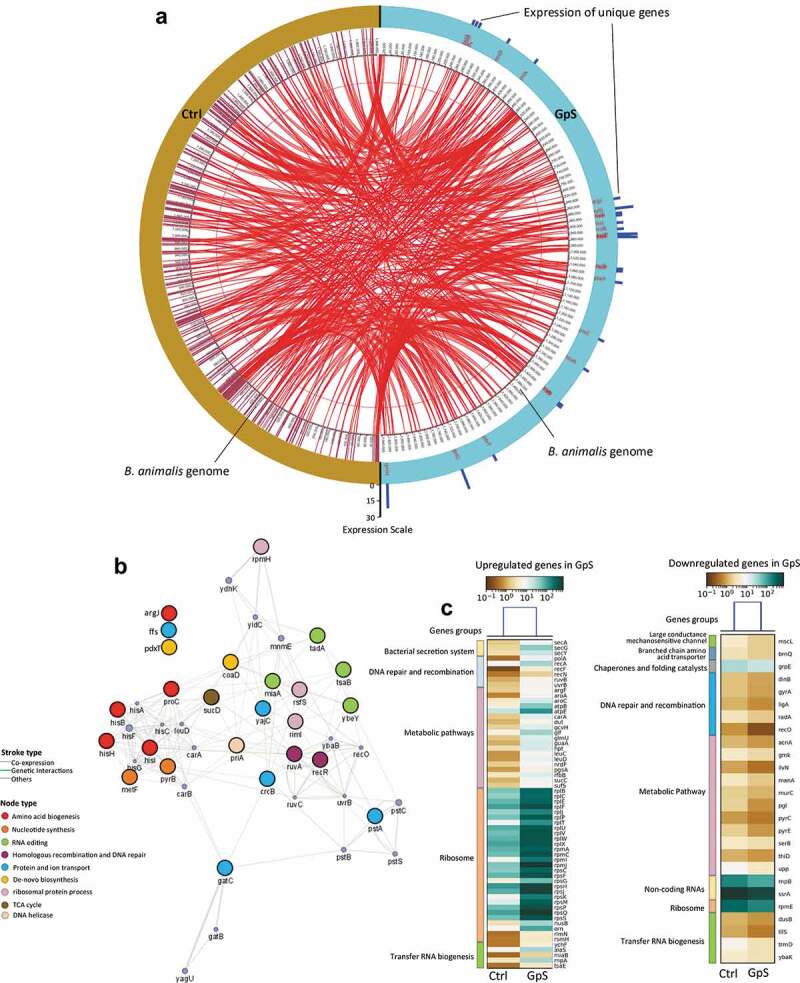
Transcriptomic analysis of *B. animalis* in the Ctrl and GpS-supplemented cultures. (a) Genes expressed both in the Ctrl and GpS cultures are displayed in the inner circle and interconnected with red lines. GpS-induced unique genes are displayed in the outmost circle, marked in blue color. The blue scale bars indicate the level of expression of the unique genes. Data was plotted with OMGenomics tool and expressed as RPKM (Reads Per Kilobase Million). (b) Network analysis of uniquely detected genes in the GpS-supplemented cultures using GeneMANIA. The nodes represent genes in the network, connected with strokes. The large nodes are the uniquely detected genes found in our dataset, whereas the small nodes are the associated genes from the database. The width of the stroke represents the strength of interaction. Weighting method is used for parameter search. Genes with same function are marked in same color. Three genes (*argJ, ffs, pdxT*) are not in the network based on their functions. (c) The upregulated and downregulated genes in tFighe GpS cultures compared to Ctrl

The data above clearly showed that GpS could stimulate the growth of *B. animalis* and enhance SCFAs/MCFAs metabolism. We went on to investigate the influence of GpS on gene expressions of *B. animalis*. The bacteria were inoculated in the growth medium in the presence and absence of GpS. Cells were harvested at a 15-h time point and subjected to RNAseq analysis. As shown in the gene map (Figure 5a and Table S1), 25 genes that were uniquely expressed in the GpS-treated RNA sample. Among the uniquely expressed genes, *rpmH, gatC, yajC, ruvA*, and *rsfS* were highly expressed. The network analysis showed that most of these uniquely expressed genes are interconnected through different cellular processes, including RNA processing, α-amino acid biosynthesis and metabolism, anion transmembrane activity, and transferase activity (Figure 5b). Also, lists of GpS-upregulated and downregulated genes are displayed in the heatmaps shown in Figure 5c and listed in Tables S2 and S3. Most of the differentially expressed genes were mapped to various metabolic pathways (Figure 5c).

### Colonization of *B. animalis* effectively reduces the number of polyps in Apc^Min/+^ mice

**Figure 6.**
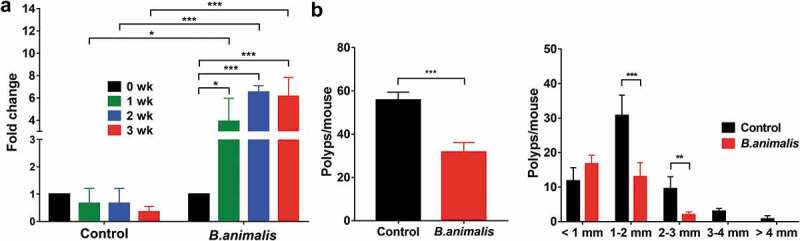
Colonization of *B. animalis* reduced the polyp number in Apc*^Min/+^* mice. (a) The presence of *B. animalis* in the fecal samples from the inoculated Apc*^Min/+^* mice, detected by qPCR. (b) Total polyps count, and the size distribution of the polyps. Ten Apc*^min/+^* mice (aged 8 weeks, male) were randomly divided into *B. animalis* treatment group and control group. Five mice were gavage with 1 × 10^9^ CFU dose of *B. animalis* weekly for three consecutive weeks to the treatment group. Control group was fed with equal volume of water. Data is presented as the mean ± SD, n = 5. Statistically significance was calculated with two-way ANOVA multiple comparison for (a) and T-test for (b). *p ≤ .05, **p ≤ .01, ***p ≤ .001

Next, we wanted to investigate whether colonization of *B. animalis* could alleviate the tumor burden in Apc*^Min/+^* mice. We challenged the Apc*^Min/+^* mice with 10^9^ live *B. animalis* cells weekly for three consecutive weeks. In parallel, five mice were gavage with saline solution as the control. A high level of *B. animalis* was readily detected in the fecal samples of the inoculated mice in the first week of the transplant and maintained a high level throughout the FMT experiment (Figure 6a). At the end of 3 weeks, the mice were euthanized for polyp count. The Apc*^Min/+^* mice colonized with *B. animalis* showed a substantial reduction in polyps by 41% compared to the control. The effect was more prominent in the medium (1–3 mm) and large (3–4 and >4 mm) size polyps (Figure 6b).

## Discussion

Our previous studies have illustrated the health benefits of the microbiota-modulating agent – GpS, in both normal and cancer-prone mouse models.^26,27,29^ One of the beneficial effects of GpS is its effectiveness in suppressing potential harmful bacteria, such as sulfur-reducing bacteria and promoting the SCFA-producing bacteria. As a result, GpS displays potent anti-inflammatory and anticancer properties in Apc*^Min/+^* mouse models. These observations lead us to the current investigation and search for a particular group(s) of commensal bacteria that might account for the anticancer effect of GpS. In this study, we first found that inoculation with fecal materials from the GpS-treated Apc*^Min/+^* mice effectively reduced the polyp burden in the untreated Apc*^Min/+^* mice. Also, a marked increase in the beneficial bacteria was detected in the feces of the FMT recipient Apc*^Min/+^* mice. These bacteria include *lactobacillus, bifidobacterium, Clostridium cluster IV*, and the SCFA-producing *Faecalibacterium prausnitzii* (Figure 2b). Many of them are well-known probiotics.

Furthermore, through the culturomic approach, we isolated 16 bacterial species from the feces of Apc/+GpS mice (Figure 3a). Out of the 16 isolates, *B. animalis* responded significantly to the growth stimulation of GpS (Figure 3). In addition to the 16 isolated bacteria, we also included two of the probiotics, *Akkermansia muciniphila* and *Lactobacillus casei* (acquired commercially) in the test. Interestingly, *Lactobacillus casei* also responded to GpS growth stimulation.

We took a great interest in *B. animalis* since the bacterium is well recognized for its probiotic function in the food industry. As a probiotic, the bacterium can produce SCFAs through the fermentation of dietary fiber. In this study, we showed that the GpS supplement increases both SCFAs and MSFAs metabolites in the cultures of *B. animalis* at different growth phases. This observation is in line with our previous finding in which high levels of serum SCFAs were significantly elevated in mice treated with GpS.^29^

To further investigate whether GpS treatment could alter the biosynthesis and metabolism of the bacteria, we looked into the transcriptome of *B. animalis* in the presence and absence of GpS using RNAseq analysis. By comparing to the Ctrl, we identified 25 genes uniquely expressed in the GpS-treated culture of *B. animalis*. These genes are mapped to various biosynthesis pathways using the KEGG Mapper (Figure 5b). For instance, on the map, *hisB, hisH, and hisI* are part of the 10-gene cluster encode steps in the histidine biosynthetic pathway.^34^ *ArgJ* encodes duel enzymes for arginine biosynthesis. *MetF* encodes 5,10-methylenetetrahydrofolate reductase, responsible for converting dUMP to dTMP for *de novo* synthesis. *PyrB* is mapped to pyrimidine biosynthesis; and *miaA, tadA, tsaB, ybeY* encode enzymes for RNA editing and synthesis.^35^ Besides playing the roles in biogenesis and biosynthesis as described above, some of the genes have different unique functions; for instance, *recR* encodes RecR protein, together with RecF and RecO proteins, forms the RecFOR complex which functions in RecA-mediated replication and homologous recombination. The *recA, F*, and *O* genes are all upregulated in GpS-cultures (Figure 5c). Another interesting gene, *ruvA*, encores part of RuvA-B DNA helicase for DNA repair and recombination.^36^ RuvB is also found upregulated on our gene list (Figure 5c and Table S2). Both the RuvA-B complex and the RecR are critical to bacterial DNA repair. The *yajC* gene encodes the smaller subunit of the preprotein translocase complex, which interacts with membrane protein SecD and SecF to coordinate protein transport and secretion across the cytoplasmic membrane in *Escherichia coli*.^37^ *PstA* encodes the subunit of the ABC transporter, and the *gatC* gene encodes a translation factor. The *coaD* encodes phosphopantetheine adenyltransferase, which is involved in coenzyme-A biosynthesis. The *coaD* gene is also a frequent target for antibacterial drug discovery.^38^ The 4.5 RNA encoded by the *ffs* gene is the RNA component of the signal recognition particle (SRP) ribonucleoprotein complex that binds to the ribosome. SRP complex involves co-translational protein secretion and requires for cell viability. Deficiency of the gene causes a dramatic loss in protein synthesis, and eventual cell death.^39^ The *rpmH* encodes ribosomal 50S ribosomal subunit protein L34. It is worth mentioning that good numbers of upregulated genes in GpS-culture are the genes encoding 50S and 30S ribosomal subunit proteins (Figure 5c and Table S2). Transcription of rRNA is an essential step in ribosome biogenesis, which is highly regulated by the external supply of nutrients or external stimuli. In our case, GpS is well served as the growth stimulus to *B. animalis* through the activations of a series of genes encoding for rRNA and various biogenesis protein molecules, as illustrated above.

GpS, the bioactive constituents of Gp, possesses many documented health benefits. One of which is the remarkably cancer-preventive properties revealed in several of our previous studies. We also demonstrated that the cancer-preventive effect of GpS might be through the modulation of the gut commensal bacteria.^27–29^ Here, for the first time, we show that the GpS-responder *B. animalis* exhibits a potent anticancer activity in Apc*^Min/+^* mice. Until now, most of the intestinal microbiota could not be easily cultured in the laboratory. For that reason, we agree that *B. animalis* might not be the only one. Still, *B. animalis* represents a group of gut commensals accounting for the cancer-preventive property of GpS.

## Conclusions

**Figure 7.**
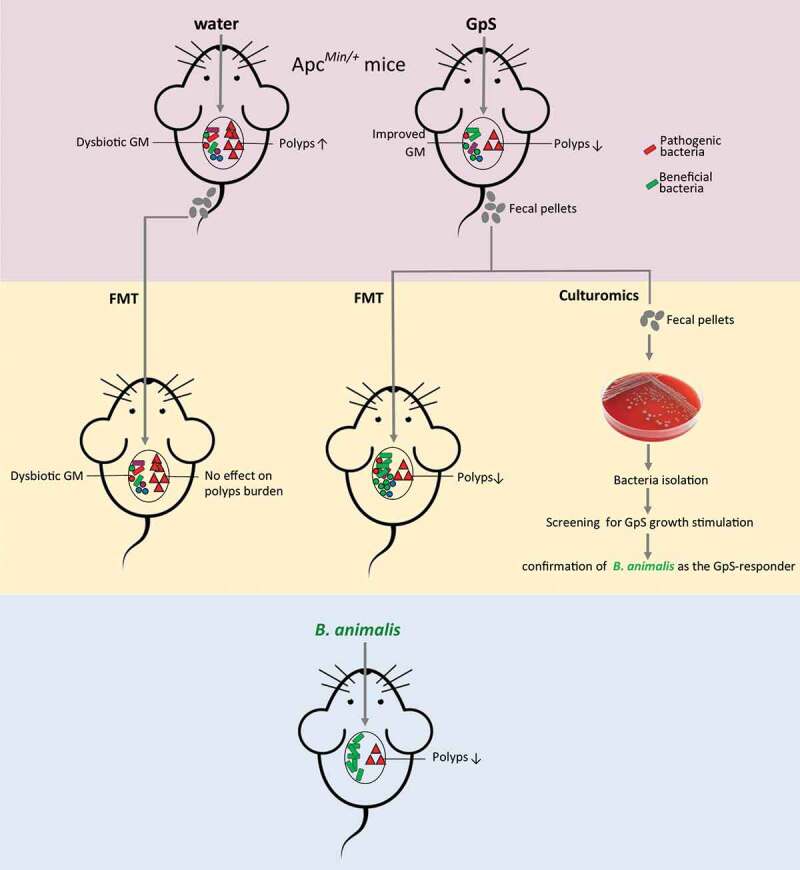
The graph summary of the mutualistic interaction between a probiotic – *B. animalis* and the prebiotic – Gp saponins in the course of cancer prevention in Apc*^Min/+^* mice

In summary, this study provides strong evidence showing how cancer-preventive activity can be achieved through the mutualistic interaction between a probiotic – *B. animalis* and the prebiotic – GpS (A graphic summary of this interaction is illustrated in Figure 7). Furthermore, the novel finding of the anticancer property of *B. animalis* may have a significant clinical implication in cancer prevention and cancer therapy in the foreseen future.

## Supplementary Material

Supplemental Material

## Data Availability

RNAseq data are deposited in the NCBI SRA (SRA: BioProject: PRJNA656358). Additional data are provided in the supplementary file.
