# Supplementary material for: Bifidobacterium animalis: the missing link for the cancer-preventive effect of Gynostemma pentaphyllum
Source: Gut Microbes. 2020 Nov 24;13(1):1847629. doi: 10.1080/19490976.2020.1847629 (PMC8381792; doi:10.1080/19490976.2020.1847629)
Supplement: Supplemental Material [file KGMI_A_1847629_SM3133.docx]

**Supplementary materials**

***Bifidobacterium animalis*, the missing link for the cancer-preventive effect of *Gynostemma pentaphyllum* saponins**

Weilin Liao#, Imran Khan#, Guoxin Huang, Shengshuang Chen, Liang Liu, Wai Kit Leong, Xiao ang Li, Jianlin Wu & W.L.Wendy Hsiao*

**Authors affiliation**

State Key Laboratory of Quality Research in Chinese Medicine, Macau University of Science and Technology, Macau, China

**Corresponding authors:** W.L. Wendy Hsiao. State Key Laboratory of Quality Research in Chinese Medicine, Macau university of science and technology, Avenida Wai Lung, Taipa, Macau SAR. E-mail: [wlhsiao@must.edu.mo](mailto:wlhsiao@must.edu.mo).

This file includes Tables S1, S2, S3, S4, S5

**Table S1: Genes uniquely expressed in *B. animalis* culture supplemented with GpS**

| **Genes** | **Ctrl** | **GpS** (RPKM) | **Encode** |
| --- | --- | --- | --- |
| *argJ* | 0 | 4.14 | Arginine biosynthesis bifunctional protein ArgJ |
| *coaD* | 0 | 2.54 | pantetheine-phosphate adenylyltransferase |
| *crcB* | 0 | 1.1 | Putative fluoride ion transporter CrcB |
| *ffs* | 0 | 1.23 | signal recognition particle 4.5S RNA |
| *gatC* | 0 | 21.34 | PTS system galactitol-specific EIIC component |
| *hisB* | 0 | 2.47 | imidazoleglycerol-phosphate dehydratase/histidinol-phosphatase |
| *hisH* | 0 | 1.7 | imidazole glycerol phosphate synthase subunit HisH |
| *hisI* | 0 | 2.59 | putative bifunctional phosphoribosyl-AMP cyclohydrolase |
| *metF* | 0 | 0.63 | 5,10-methylenetetrahydrofolate reductase |
| *miaA* | 0 | 1.32 | tRNA dimethylallyltransferase |
| *pdxT* | 0 | 3.79 | Pyridoxal 5'-phosphate synthase subunit PdxT |
| *priA* | 0 | 1.76 | primosome factor N |
| *proC* | 0 | 3.33 | pyrroline-5-carboxylate reductase |
| *pstA* | 0 | 1.11 | phosphate ABC transporter membrane subunit PstA |
| *pyrB* | 0 | 2.17 | aspartate carbamoyltransferase catalytic subunit |
| *recR* | 0 | 1.92 | DNA repair protein RecR |
| *rimI* | 0 | 0.36 | ribosomal-protein-S18-alanine N-acetyltransferase |
| *rpmH* | 0 | 27.77 | 50S ribosomal subunit protein L34 |
| *rsfS* | 0 | 17.75 | ribosomal silencing factor RsfS |
| *ruvA* | 0 | 19.93 | Holliday junction branch migration complex subunit RuvA |
| *sucD* | 0 | 5.71 | succinyl-CoA synthetase subunit alpha |
| *tadA* | 0 | 0.17 | tRNA adenosine(34) deaminase |
| *tsaB* | 0 | 1.09 | tRNA threonylcarbamoyladenosine biosynthesis protein TsaB |
| *yajC* | 0 | 20.38 | Sec translocon accessory complex subunit YajC |
| *ybeY* | 0 | 4.69 | Endoribonuclease YbeY |

p.s.: RPKM: Reads Per Kilobase Million.

**Table S2: List of upregulated genes**

| **Functional group** | **Gene** | **Ctrl** (RPKM) | **GpS** (RPKM) | **Fold change (%)** | **Gene functions** |
| --- | --- | --- | --- | --- | --- |
| Bacterial secretion system | *secA* | 1.66 | 7.73 | 365.66 | Protein translocation ATPase |
|  | *secG* | 2.17 | 44.34 | 1943.32 | Sec translocon subunit SecG |
|  | *secY* | 2.7 | 19.53 | 623.33 | Sec translocon subunit SecY |
| DNA repair and recombination | *polA* | 0.42 | 9.45 | 2150.00 | DNA polymerase I |
|  | *recA* | 7.75 | 33.28 | 329.42 | DNA recombination |
|  | *recF* | 0.19 | 4.38 | 2205.26 | DNA repair protein RecF |
|  | *recN* | 0.47 | 1.92 | 308.51 | DNA repair protein RecN |
|  | *ruvB* | 1.01 | 6.8 | 573.27 | Holliday junction branch migration complex subunit RuvB |
|  | *uvrB* | 1.53 | 6.75 | 341.18 | Excision nuclease subunit B |
| Metabolic pathways | *argF* | 1.82 | 7.54 | 314.29 | CP4-6 prophage; ornithine carbamoyltransferase ArgF |
|  | *aroA* | 0.58 | 8.87 | 1429.31 | 3-phosphoshikimate 1-carboxyvinyltransferase |
|  | *aroC* | 3.74 | 15.97 | 327.01 | Chorismate synthase |
|  | *atpB* | 5.15 | 48.02 | 832.43 | ATP synthase Fo complex subunit a |
|  | *atpE* | 21.04 | 125.99 | 498.81 | ATP synthase Fo complex subunit c |
|  | *carA* | 1.64 | 12.95 | 689.63 | carbamoyl phosphate synthetase subunit alpha |
|  | *dut* | 1.15 | 6.25 | 443.48 | Deoxyuridine triphosphatase |
|  | *gcvH* | 1.97 | 13.54 | 587.31 | Glycine cleavage system H protein |
|  | *glf* | 5.92 | 35.31 | 496.45 | UDP-galactopyranose mutase |
|  | *glmU* | 1.56 | 13.25 | 749.36 | Bifunctional protein GlmU |
|  | *guaA* | 1.04 | 15.61 | 1400.96 | GMP synthetase |
|  | *hpt* | 2.63 | 15.88 | 503.80 | hypoxanthine phosphoribosyltransferase |
|  | *leuC* | 0.77 | 6.99 | 807.79 | 3-isopropylmalate dehydratase subunit LeuC |
|  | *leuD* | 0.79 | 10.66 | 1249.37 | 3-isopropylmalate dehydratase subunit LeuD |
|  | *nrdF* | 1.82 | 8.66 | 375.82 | Ribonucleoside-diphosphate reductase 2 subunit beta |
|  | *pgsA* | 0.83 | 5.64 | 579.52 | Phosphatidylglycerophosphate synthase |
|  | *rfbB* | 3.18 | 12.81 | 302.83 | dTDP-glucose 4,6-dehydratase 1 |
|  | *sucC* | 1.13 | 8.95 | 692.04 | Succinyl-CoA synthetase subunit beta |
|  | *sufS* | 1.7 | 7.3 | 329.41 | L-cysteine desulfurase |
| Ribosomal structure | *rplB* | 51.17 | 270.9 | 429.41 | 50S ribosomal subunit protein L2 |
|  | *rplC* | 38.99 | 256.58 | 558.07 | 50S ribosomal subunit protein L3 |
|  | *rplE* | 64.62 | 271.03 | 319.42 | 50S ribosomal subunit protein L5 |
|  | *rplF* | 93.5 | 417.39 | 346.41 | 50S ribosomal subunit protein L6 |
|  | *rplJ* | 36.72 | 161.5 | 339.81 | 50S ribosomal subunit protein L10 |
|  | *rplP* | 55.61 | 338.18 | 508.13 | 50S ribosomal subunit protein L16 |
|  | *rplT* | 8.79 | 125.39 | 1326.51 | 50S ribosomal subunit protein L20 |
|  | *rplU* | 77.77 | 337.16 | 333.53 | 50S ribosomal subunit protein L21 |
|  | *rplV* | 58.5 | 408.93 | 599.03 | 50S ribosomal subunit protein L22 |
|  | *rplW* | 70.91 | 372.33 | 425.07 | 50S ribosomal subunit protein L23 |
|  | *rplX* | 91.21 | 394.47 | 332.49 | 50S ribosomal subunit protein L24 |
|  | *rpmA* | 71.79 | 296.4 | 312.87 | 50S ribosomal subunit protein L27 |
|  | *rpmC* | 59.64 | 387.1 | 549.06 | 50S ribosomal subunit protein L29 |
|  | *rpmI* | 6.33 | 203.5 | 3114.85 | 50S ribosomal subunit protein L35 |
|  | *rpmJ* | 49.74 | 662.25 | 1231.42 | 50S ribosomal subunit protein L36 |
|  | *rpsC* | 46.13 | 264.08 | 472.47 | 30S ribosomal subunit protein S3 |
|  | *rpsF* | 67.03 | 387.89 | 478.68 | 30S ribosomal subunit protein S6 |
|  | *rpsG* | 4.59 | 59.07 | 1186.93 | 30S ribosomal subunit protein S7 |
|  | *rpsH* | 114.36 | 514.99 | 350.32 | 30S ribosomal subunit protein S8 |
|  | *rpsJ* | 73.84 | 521.01 | 605.59 | 30S ribosomal subunit protein S10 |
|  | *rpsK* | 6.43 | 181.16 | 2717.42 | 30S ribosomal subunit protein S11 |
|  | *rpsM* | 14.29 | 302.62 | 2017.70 | 30S ribosomal subunit protein S13 |
|  | *rpsP* | 57.9 | 447.38 | 672.68 | 30S ribosomal subunit protein S16 |
|  | *rpsQ* | 94.14 | 630.97 | 570.25 | 30S ribosomal subunit protein S17 |
|  | *rpsS* | 56.13 | 369.58 | 558.44 | 30S ribosomal subunit protein S19 |
| Ribosome biogenesis | *nusB* | 2.9 | 33.15 | 1043.10 | Transcription antitermination protein NusB |
|  | *orn* | 7.43 | 88.32 | 1088.69 | Oligoribonuclease |
|  | *rlmN* | 0.45 | 3.83 | 751.11 | Dual-specificity RNA methyltransferase RlmN |
|  | *rsmH* | 0.51 | 3.56 | 598.04 | Ribosomal RNA small subunit methyltransferase H |
|  | *ychF* | 0.49 | 4.7 | 859.18 | Redox-responsive ATPase YchF |
| Transfer RNA biogenesis | *alaS* | 6.55 | 31.82 | 385.80 | Alanine--tRNA ligase |
|  | *miaB* | 0.36 | 2 | 455.56 | Isopentenyl-adenosine A37 tRNA methylthiolase |
|  | *rnpA* | 3.03 | 31.26 | 931.68 | RNase P protein component |
|  | *tsaE* | 0.43 | 3.59 | 734.88 | tRNA threonylcarbamoyladenosine biosynthesis protein TsaE |

p.s.: RPKM: Reads Per Kilobase Million.

**Table S3: List of downregulated genes.**

| **Functional group** | **Gene** | **Control** (RPKM) | **GpS** (RPKM) | **Fold change (%)** | **Gene functions** |
| --- | --- | --- | --- | --- | --- |
| Large conductance mechanosensitive channel | *mscL* | 13.47 | 4.99 | -62.9 | large conductance mechanosensitive channel |
| Branched chain amino acid transporter | *brnQ* | 8.7 | 4.8 | -44.8 | branched chain amino acid transporter BrnQ |
| Chaperones and folding catalysts | *grpE* | 194.68 | 119.68 | -38.5 | nucleotide exchange factor GrpE |
| DNA repair and recombination | *dinB* | 3.49 | 1.94 | -44.4 | DNA polymerase IV |
|  | *gyrA* | 3.52 | 1.81 | -48.6 | DNA gyrase subunit A |
|  | *ligA* | 2.5 | 1.31 | -47.6 | DNA ligase |
|  | *radA* | 9.86 | 4.23 | -57.1 | DNA recombination protein |
|  | *recO* | 1.5 | 0.29 | -80.7 | DNA repair protein RecO |
| Metabolic Pathway | *acnA* | 2.8 | 1.61 | -42.5 | aconitate hydratase 1 |
|  | *gmk* | 12.86 | 6.75 | -47.5 | guanylate kinase |
|  | *ilvN* | 1.95 | 0.75 | -61.5 | acetohydroxy acid synthase I subunit IlvN |
|  | *manA* | 10.63 | 4.17 | -60.8 | mannose-6-phosphate isomerase |
|  | *murC* | 4.92 | 2.71 | -44.9 | UDP-N-acetylmuramate--L-alanine ligase |
|  | *pgl* | 5.28 | 1.5 | -71.6 | 6-phosphogluconolactonase |
|  | *pyrC* | 1.71 | 0.88 | -48.5 | dihydroorotase |
|  | *pyrE* | 4.85 | 1.62 | -66.6 | orotate phosphoribosyltransferase |
|  | *serB* | 11.15 | 7.76 | -30.4 | phosphoserine phosphatase |
|  | *thiD* | 2.44 | 1.63 | -33.2 | bifunctional hydroxymethylpyrimidine kinase |
|  | *upp* | 33.65 | 11.17 | -66.8 | uracil phosphoribosyltransferase |
| Non-coding RNAs | *rnpB* | 1939.38 | 857.51 | -55.8 | RNase P catalytic RNA component |
|  | *ssrA* | 12772.48 | 8675.99 | -32.0 | tmRNA |
| Ribosome | *rpmE* | 3645.41 | 1876.42 | -48.5 | 50S ribosomal subunit protein L31 |
| Transfer RNA biogenesis | *dusB* | 2.58 | 1.57 | -39.1 | tRNA-dihydrouridine synthase B |
|  | *tilS* | 2.1 | 0.93 | -55.7 | tRNA(Ile)-lysidine synthetase |
|  | *trmD* | 26.74 | 8.95 | -66.5 | tRNA m(1)G37 methyltransferase |
|  | *ybaK* | 13.24 | 8.99 | -32.1 | Cys-tRNA(Pro) and Cys-tRNA(Cys) deacylase |

p.s.: RPKM: Reads Per Kilobase Million.

| Ingredients | Grams per liter |
| --- | --- |
| Acid Hydrolysate of Casein | 0.24 |
| Agar | 13 |
| Beef heart | 38.4 |
| Calf brain | 30.76 |
| Dextrose | 0.87 |
| Dipotassium Phosphate | 1.42 |
| Disodium phosphate | 0.43 |
| Magnesium Sulfate Heptahydrate | 0.024 |
| Pancreatic digest of casein | 1.7 |
| Piapiac digest of soybean meal | 0.3 |
| Proteose peptone | 2.16 |
| Sodium chloride | 1.58 |
| Sodium Pyruvate | 0.14 |
| Soluble Starch | 0.24 |
| Yeast Extract | 0.24 |

**Table S4: The formulation of the basic bacterial growth medium**

**Table S5: Primers sequences for qPCR assays**

| Bacteria | Primer | Primer sequences (5' to 3') | Reference |
| --- | --- | --- | --- |
| Total bacteria | UniF340 | ACTCCTACGGGAGGCAGCAGT | [1] |
|  | UniR514 | ATTACCGCGGCTGCTGGC |  |
| *Firmicutes* | 928F-Firm | TGAAACTYAAAGGAATTGACG | [2] |
|  | 1040FirmR | ACCATGCACCACCTGTC |  |
| *Bacteroidetes* | 798cfbF | CRAACAGGATTAGATACCCT | [2] |
|  | cfb967R | GGTAAGGTTCCTCGCGTAT |  |
| *Bacteroides* | BactF296 | GAGAGGAAGGTCCCCCAC | [3] |
|  | BactR412 | CGCTACTTGGCTGGTTCAG |  |
| *Lactobacillus* | LabF362 | AGCAGTAGGGAATCTTCCA | [4] |
|  | LabR677 | CACCGCTACACATGGAG |  |
| *Bifidobacterium* | BifF | GCGTGCTTAACACATGCAAGTC | [4] |
|  | BifR | CACCCGTTTCCAGGAGCTATT |  |
| *Clostridium* Cluster IV | Clep866mF | TTAACACAATAAGTWATCCACCTGG | [5] |
|  | Clep1240mR | ACCTTCCTCCGTTTTGTCAAC |  |
| *Faecalibacterium prausnitzii* | Fprau223F  Fprau420R | GATGGCCTCGCGTCCGATTAG  CCGAAGACCTTCTTCCTCC | [6] |

**References:**

1. Croswell A, Amir E, Teggatz P, Barman M, Salzman NH. Prolonged impact of antibiotics on intestinal microbial ecology and susceptibility to enteric Salmonella infection. Infect. Immun. 2009;77:2741–53.

2. Bacchetti De Gregoris T, Aldred N, Clare AS, Burgess JG. Improvement of phylum- and class-specific primers for real-time PCR quantification of bacterial taxa. J. Microbiol. Methods. 2011;86:351–6.

3. Guo X, Xia X, Tang R, Zhou J, Zhao H, Wang K. Development of a real-time PCR method for Firmicutes and Bacteroidetes in faeces and its application to quantify intestinal population of obese and lean pigs. Lett. Appl. Microbiol. England; 2008;47:367–73.

4. Penders J, Thijs C, Vink C, Stelma FF, Snijders B, Kummeling I, et al. Factors influencing the composition of the intestinal microbiota in early infancy. Pediatrics. United States; 2006;118:511–21.

5. Ramirez-Farias C, Slezak K, Fuller Z, Duncan A, Holtrop G, Louis P. Effect of inulin on the human gut microbiota: stimulation of Bifidobacteriumadolescentis and Faecalibacterium prausnitzii. Br J Nutr. 2009;101.

6. Bartosch S, Fite A, Macfarlane GT, McMurdo MET. Characterization of bacterial communities in feces from healthy elderly volunteers and hospitalized elderly patients by using real-time PCR and effects of antibiotic treatment on the fecal microbiota. Appl. Environ. Microbiol. American Society for Microbiology; 2004;70:3575–81.
